# A Sensitive Electrochemical Cholinesterase-Inhibiting Biosensor for Organophosphorus Pesticides Based on Ti_3_C_2_T_X_ MXene Quantum Dots

**DOI:** 10.3390/bios15090575

**Published:** 2025-09-02

**Authors:** Nisha Makani, Jett Wu, Jose Florentino, Cecilia F. Chafin, Bhoj Gautam, Shirley Chao, Shubo Han

**Affiliations:** 1 Department of Chemistry, Physics and Materials Science, Fayetteville State University, Fayetteville, NC 28301, USA; npatelmakani@uncfsu.edu (N.M.);; 2 Department of Biological and Forensic Sciences, Fayetteville State University, Fayetteville, NC 28301, USAschao@uncfsu.edu (S.C.)

**Keywords:** Ti_3_C_2_T_x_ MXene, electrochemical biosensor, quantum dots, cholinesterase inhibitor, organophosphorus pesticides

## Abstract

Organophosphorus pesticides (OPs) pose significant environmental and health risks due to their widespread use and toxicity, primarily by inhibiting acetylcholinesterase. Traditional detection methods are often slow and costly, highlighting the urgent need for advanced, sensitive, and accessible technologies. This study developed a highly sensitive electrochemical cholinesterase-inhibiting biosensor for OP pesticides, utilizing Ti_3_C_2_T_x_ MXene Quantum Dots (MQDs), which was synthesized via a hydrothermal method. The biosensor’s performance was characterized using electrochemical impedance spectroscopy, differential pulse voltammetry (DPV), and cyclic voltammetry. DPV proved to be the optimal technique, exhibiting an ultralow detection limit of 1 × 10^−17^ M and a wide linear range (10^−14^–10^−8^ M) for chlorpyrifos (a model OP) with an estimated inhibition constant of 62 nM. The biosensor demonstrated high selectivity for OPs (chlorpyrifos, acephate, glyphosate) over a non-target pyrethroid (permethrin), confirmed by distinct electrochemical signatures and compared to in vitro cholinergic activity assays in bean beetle homogenates. The enhanced performance is attributed to the high surface-to-volume ratio, quantum confinement effects, and superior conductivity of the MQDs, as well as the robust enzyme immobilization facilitated by glutaraldehyde cross-linking and a chitosan matrix. This work presents a promising platform for rapid, sensitive, and selective detection of OP pesticides, with potential applications in environmental monitoring and public health protection.

## 1. Introduction

Organophosphorus pesticides (OPs) are extensively used globally in agriculture and various other sectors, posing significant environmental and health risks due to their widespread presence and inherent toxicity to both non-target organisms and humans [1,2]. These compounds exert harmful effects primarily by inhibiting acetylcholinesterase (AChE), an enzyme crucial for nervous system function, leading to an excessive accumulation of acetylcholine and subsequent neurological overstimulation. This disruption can manifest in acute symptoms ranging from neurological impairments to respiratory failure and even death, with chronic low-level exposure also raising concerns for long-term health. Timely intervention and proactive monitoring are critical to prevent irreversible neurological damage, respiratory failure, and death, while also reducing the global public health burden of these exposures [3,4,5]. Conventional laboratory methods, such as High-Performance Liquid Chromatography (HPLC) or Gas Chromatography (GC) coupled with Mass Spectrometry (MS), are widely recognized for their high accuracy and specificity in pesticide detection. However, these established techniques often come with considerable operational costs, demand extensive and time-consuming sample preparation and complex rapid on-site detection and necessitate the expertise of highly skilled personnel [6,7,8]. Biosensors offer a complementary approach to conventional laboratory-based techniques by simplifying or eliminating complex sample preparation steps, thereby addressing some of the inherent limitations of traditional methods [6,9,10,11]. This underscores an urgent imperative for developing advanced, sensitive, and accessible detection technologies to effectively manage these widespread contaminants and protect public health and the environment [12,13,14,15].

Electrochemical biosensors present a highly promising alternative to conventional analytical methods for pesticide detection, offering significant advantages such as cost-effectiveness, portability, rapid response times, and minimal sample preparation, making them ideal for on-site and in situ applications [6,7,16,17]. For cholinesterase inhibitor pesticides, the primary detection mechanism relies on enzyme inhibition, where AChE biosensors quantify the degree of enzyme inhibition, which directly correlates with pesticide concentration. While biosensor technology faces challenges such as matrix interference and surface fouling, the integration of advanced nanomaterials, including carbon nanotubes, gold nanoparticles, and quantum dots, has revolutionized their performance, substantially improving sensitivity, selectivity, stability, and suitability for real-world analysis [18,19,20,21]. Carbon nanotubes (CNTs) enhance electrical conductivity and provide large surface areas for enzyme immobilization, thereby improving sensitivity [19,20]. Gold nanoparticles (AuNPs) facilitate efficient electron transfer and offer biocompatible surfaces for enzyme conjugation, resulting in enhanced stability and selectivity [18]. Similarly, quantum dots (QDs) introduce quantum confinement effects that promote rapid electron transport and signal amplification, enabling detection at ultra-trace levels [21]. These nanostructured materials not only improve analytical performance but also extend operational stability, making biosensors increasingly suitable for real-world applications such as food safety monitoring, environmental surveillance, and point-of-care diagnostics. As a result, the integration of nanomaterials into electrochemical biosensors has revolutionized their applicability, pushing the boundaries of detection sensitivity, selectivity, and robustness for monitoring pesticide contamination in diverse and complex environments.

MXenes, an emerging class of two-dimensional (2D) transition metal carbides, nitrides, and carbonitrides, are particularly promising for developing high-sensitivity, high-stability, and multifunctional biosensors due to their unique layered structure and outstanding electrochemical properties [22,23,24,25,26,27,28,29]. 2D MXenes are a family of materials with the general formula M_n+1_X_n_T_x_ (n = 1–3), where M represents a group 13–16 transition metal (e.g., Ti, V, Nb), X is carbon and/or nitrogen, and T denotes surface terminal groups such as –OH, –F, or –Cl. They are typically synthesized by selectively removing group 13 or 14 elements (commonly Al or Si, collectively referred to as A) from their parent MAX phases, which are layered ternary carbides or nitrides. The first MXene, Ti_3_C_2_T_x_, was reported by Gogotsi and co-workers, who produced it by etching Al layers from Ti_3_AlC_2_ using hydrofluoric acid (HF) [30]. Since then, Ti_3_C_2_T_x_ has become the most widely studied MXene due to its exceptional electrical conductivity, large specific surface area, and highly tunable surface functionalities (−O, −F, −OH), achieved through HF or alternative etching methods [31]. The high conductivity accelerates electron transfer kinetics, the expansive surface area enables high-density bioreceptor immobilization, and the tunable functional groups allow precise bio-conjugation, making them ideal as immobilization matrices for enzymes [27,32,33,34].

Notably, Ti_3_C_2_T_x_ MXene QDs (MQDs) inherit and enhance these advantages through powerful quantum confinement effects, exhibiting unparalleled surface-to-volume ratios, abundant active edge atoms, and superior photostability, biocompatibility, and dispersibility, making them ideal for biosensing [35,36,37]. This atomic-level tunability, combined with rapid electron transfer, positions MXenes and MQDs as transformative materials for next-generation biosensors, overcoming the limitations of conventional nanomaterials in sensitivity, selectivity, and functionalization, pushing the detection limits beyond traditional materials [36,38].

In this work, Ti_3_C_2_T_x_ MQDs were synthesized by a hydrothermal method and characterized by atomic force microscopy (AFM), scanning electron microscopy (SEM), X-ray diffraction (XRD), UV–Vis absorption, and photoluminescence. An electrochemical cholinesterase-inhibiting biosensor was developed based on the synthesized Ti_3_C_2_T_x_ MQDs for detection of OP pesticides, a first-of-its-kind application for this material in the literature. The sensor performance was validated by electrochemical impedance spectroscopy (EIS), differential pulse voltammetry (DPV), and cyclic voltammetry (CV). This biosensor confers unprecedented analytical performance, most notably an ultralow detection limit of 1 × 10^−17^ M for chlorpyrifos (CPS), coupled with a wide linear dynamic range spanning from 10^−14^ to 10^−8^ M. We demonstrated enhanced performance of the MQD-based biosensor in monitoring the cholinergic activity of chlorpyrifos, acephate, and glyphosate over a non-target pyrethroid (permethrin) compared with cholinergic activity assays in bean beetle homogenates. This work could contribute to the development of the OP electrochemical biosensor discipline by demonstrating that Ti_3_C_2_T_x_ MQDs, combined with optimized enzyme immobilization strategies, can enable ultratrace, selective, and stable detection of organophosphorus pesticides, thereby advancing sensitive, rapid, and field-deployable monitoring platforms for these widespread contaminants to protect public health and the environment.

## 2. Materials and Methods

### 2.1. Chemicals and Reagents

AChE from electrophorus electricus (500 units), acetylthiocholine iodide (ACTI), chitosan (CS), 50% glutaraldehyde (GA), chlorpyrifos (CPS), acephate (ACE), glyphosate (GLY), permethrin (PERM), 5,5′-Dithiobis(2-nitrobenzoic acid) (DTNB), bovine serum albumin (BSA), titanium aluminum carbide MAX phase, sodium hydroxide (NaOH), and glacial acetic acid (HAc) were obtained from Sigma-Aldrich (St. Louis, MO, USA). All other chemicals, including lithium fluoride (LiF, ≥99.98% purity), hydrochloric acid (HCl, 37%, ACS reagent grade), dibasic sodium phosphate (Na_2_HPO_4_), monobasic sodium phosphate (NaH_2_PO_4_), and all the solvents, were purchased from Fisher Scientific (Hampton, NH, USA).

### 2.2. Characterization

The synthesized MXene sheets and MQDs were thoroughly characterized using a range of analytical techniques. XRD was conducted on a Rigaku diffractometer Miniflex 600 X-ray diffractometer (Rigaku Corporation, Tokyo, Japan) equipped with a Cu Kα line (λ = 1.540 Å) radiation source (40 kV, 15 mA), scanning over a 2θ range of 5° to 60° at a scan rate of 1°/min and a step size of 0.02°.

SEM images were acquired using a JEOL Field Emission SEM (FESEM) (Peabody, MA, USA) operated at 20 kV. A magnification of 3500× was used to investigate the surface morphology of the samples.

Topographic images of tapping mode AFM were taken using a Keysight 5500 Atomic Force Microscope (Keysight Technologies, Inc., Colorado Springs, CO, USA) with a resolution of 512 points × 512 lines and a scanning rate of 1 line/s. A Bruker’s Sharp Nitride Lever probe, SNL-10, with a normal frequency 65 kHz and a normal spring constant of 0.35 N/m, was used in the scanning (Bruker AFM Probes, Camarillo, CA, USA). The data were processed using the Gwyddion 2.56 software to extract the topographical features and analyze the distributions of the grain characteristics.

UV–Vis absorption and photoluminescence (emission) spectra were recorded using a Horiba Duetta UV–Vis spectrometer (Piscataway, NJ, USA). Measurements were carried out in a quartz cuvette over the wavelength range of 300–750 nm under ambient conditions. Baseline correction was performed using deionized (DI) water as the blank solvent, and all spectra were acquired with appropriate resolution and integration time settings.

Time-resolved photoluminescence (PL) measurements of Ti_3_C_2_T_x_ MQDs were carried out using a LifeSpec II, F980 spectrometer (Edinburgh Instruments Ltd., Livingston, UK), equipped with a 400 nm diode laser excitation source (pulse width: 500 ns, repetition rate: 1 MHz). The average PL lifetimes were extracted by fitting the decay curves using a multi-exponential model with tail fitting, employing a nonlinear least-squares fitting algorithm.

### 2.3. Electrochemical Measurement

The electrochemical system and electrodes were obtained from CH Instruments (Austin, TX, USA): EIS, DPV, and CV measurements were performed by CHI 760E Electrochemical Workstation with a three-electrode system: The biosensor was built on a CHI104 3 mm diameter Glassy Carbon electrode as the working electrode, a 7 mm^2^ electrochemically active surface for the designed biosensor, CHI 111 saturated Ag/AgCl as the reference electrode, and CHI115 Platinum Wire as the counter electrode.

EIS was performed with the following parameters: Technique, IMP-AC Impedance. Initial potential, 0.5 V. Frequency, 1–100,000 Hz. Amplitude, 5 mV. The obtained Nyquist plots were fitted with EIS Spectrum Analyser to estimate the correct charge-transfer resistance.

DPV was performed with the following parameters: Initial potential, −0.2 V. Final potential, 1 V. Potential Increment, 4 mV. Amplitude, 50 mV. Pulse width, 50 ms. Pulse Period, 500 ms. Pulse width, 50 ms.

CV was performed with the following parameters: Initial potential, −0.2 V. Final Potential, 1 V. San Rate, 25 mV/s.

Unless declared otherwise, all electrochemical measurements were carried out in phosphate-buffered saline (PBS, pH~7.4) at 25 °C.

### 2.4. Preparation of Solutions

*LiF/HCl etching solution*: In a polyethylene container, 1.6 g LiF was added to 20 mL of 9 M HCl in polyethylene container (resistant to HF corrosion). The mixture was stirred continuously at 40 °C for 30 min under nitrogen purge until LiF fully dissolved.

*PBS (pH~7.4)*: (a). Dissolve 27.6 g NaH_2_PO_4_·H_2_O in 1 L distilled water to prepare Solution A (0.2 M NaH_2_PO_4_). (b). Dissolve 53.65 g Na_2_HPO_4_·7H_2_O in 1 L distilled water to prepare Solution B (0.2 M Na_2_HPO_4_). (c). Mix 195 mL Solution A and 805 mL Solution B, adding 8.5 g NaCl to prepare 0.1 M PBS (pH~7.4). (d). Adjust the pH to 7.4 with a 1 M NaOH solution. (e). Adjust the final volume to 1 L with distilled water. PBS was further used as the supporting electrolyte in the measurement of electrochemical biosensor characterization.

*HAc solution (1%)*: HAc solution (1%) was prepared by water-diluting glacial acetic acid, which was obtained from Sigma-Aldrich.

*AChE solution*: AChE from Electrophorus electricus (500 units) was defrosted in an ice bath over a 30 min interval, and the entire 30 μL sample was diluted in 1 mL of the PBS buffer. The prepared solution was stored in refrigerator at 2–8 °C up to 1 week.

*BSA solution*: BSA purified 10% solution was diluted 10 times with PBS. The 1% BSA was used to stabilize AChE during reactions, enhancing the signals of the prepared biosensor. A 1% BSA solution should be stored at 2–8 °C in refrigerator to best preserve its stability and prevent microbiological activity. The solution should be kept in a tightly closed container and protected from light. Before use, check that the solution is clear and free of particulates or flocculent material; any color change or cloudiness may indicate deterioration.

*CS solution (0.2% w/v, pH 6.0)*: A 0.2% CS solution was meticulously prepared by combining 0.2 g of dry powdered CS with 100 mL of a 1% acetic acid solution. The 1% acetic acid solution was developed by diluting 1 mL of glacial acetic acid to a final volume of 100 mL in a volumetric flask. CS solution was stored in tightly closed containers between 2–8 °C in refrigerator. For best results, always protect the solution from light and avoid repeated freeze–thaw cycles.

*GA solution (2.5%)*: Freshly prepare: a 2.5% GA solution by mixing 1 mL of 50% GA with 19 mL of PBS buffer to prepare for the enzyme cross-linking and immobilization.

*Preparation of OPs and the control solutions*: First, 1 mM stock solutions of the selected OPs (CPS, ACE, GLY) and the control, PERM, were prepared in ethanol. Working solutions of different concentrations were diluted from the stock solutions with PBS solutions. Ensure the final concentration ethanol in the assay does not exceed 0.01% to avoid interference with AChE activity. All the solutions should be protected from light and stored at −20 °C until use to prevent degradation.

### 2.5. Synthesis of Ti_3_C_2_T_x_ MXene 2D Sheets and MQDs

Ti_3_C_2_T_x_ *MXene 2D Sheets*: The LiF/HCl etching method was employed to synthesize Ti_3_C_2_T_x_ MXene 2D sheets [39,40]. Specifically, 2.9 g of LiF powder was dissolved in 100 mL of HCl in a Nalgene wide-mouth bottle. This mixture was placed in an oil bath to maintain a uniform temperature. After allowing the etchant solution to stabilize for 10 min, 1 g of Ti_3_AlC_2_ MAX phase was slowly added to the solution. The temperature was then raised to 70 °C, and the reaction was allowed to proceed for 7 days. Following etching, the temperature was reduced, and the mixture was subjected to sequential washing, initially with HCl, followed by deionized (DI) water until the pH of the supernatant reached approximately 6. The resulting material was then dried in an oven at 60 °C for 24 h. The dried Ti_3_C_2_T_x_ MXene powder was subsequently used for the synthesis of Ti_3_C_2_T_x_ MQDs.

*MQDs*: A hydrothermal method was employed for the synthesis of QDs [41,42]. Exfoliated Ti_3_C_2_T_x_ MXene 2D sheets were first dispersed in 10 mL of deionized (DI) water. Ammonia was then added dropwise while continuously monitoring the pH until it reached 10. The resulting dispersion was transferred into a Teflon-lined stainless-steel autoclave and heated at 100 °C for 6 h in an oven. After the hydrothermal treatment, the product was allowed to cool to room temperature. The resulting suspension was centrifuged, and the supernatant, which appeared yellowish, was carefully decanted. This solution was further purified via vacuum filtration using a membrane filter with a pore size of 0.02 µm. The filtered QD solution was then characterized and subsequently used in the fabrication of an electrochemical biosensor.

### 2.6. Preparation of Ti_3_C_2_T_x_ MQD-Based Electrochemical Biosensor

#### 2.6.1. GCE Pretreatment

*GCE Cleaning*: To ensure the GCE is free of contaminants, the GCE was first rinsed thoroughly with distilled water, followed by methanol, then the electrode was gently wiped dry with a clean, lint-free lab tissue.

*Polishing the GCE*: A nylon polishing pad was placed onto a flat glass plate. The pad was moistened with distilled water. Then, 0.3 µm alumina suspension was dropped onto the wet pad. The GCE was placed face down on the pad and polished using a figure-eight motion for 2 min. Gentle but consistent pressure was applied to avoid damaging the electrode surface. During this process, the GCE was rotated 90° periodically to ensure even wear across the surface. A mirror-like surface was achieved after this polishing was finished. To remove all remaining particles, a final cleaning and drying was processed after polishing. The electrode was sonicated in distilled water, methanol, or acetone for up to 5 min, then rinsed again and allowed to air dry at room temperature.

*Electrochemical Activation*: CV scan in 0.1 M H_2_SO_4_ was performed by scanning the potential between −0.5 V and +1.5 V at a rate of 100 mV/s for 20 cycles. This process generated oxygen-containing surface functional groups (OxSFGs) on the GCE, which can enhance adsorption and electron transfer. After the activation, the electrode was rinsed with DI water and dried under nitrogen.

*Important Precautions*: The pretreated GCE surface should not be touched with hands or sharp objects. Overheating or excessive pressure should be avoided during polishing, as this may damage the electrode.

#### 2.6.2. Ti_3_C_2_T_x_ MQD-Based Electrochemical Biosensor Prepared on the Electrochemical Activated GCE Surface

*MQD Anchoring on GCE*: MQD was mixed with CS solution (0.2% *w*/*v*, pH 6.0) to create a composite suspension. Drop-cast the metal oxide-CS composite onto the freshly activated GCE surface and allow it to dry at room temperature.

*Immobilization of AChE*: AChE was immobilized on the MQD-anchored GC surface by the following steps: (a). AChE solution was mixed with BSA (AChE 15 mg/mL, BSA, 0.9%), which was used as a stabilizer and spacer molecule. (b). AChE/BSA was mixed with freshly prepared GA at a 2:1 ratio to facilitate crosslinking and immobilization onto the MQDs surface. (c). Drop-cast the AChE/BSA/GA solution onto the MQDs-anchored GCE surface and incubate for 2 h to allow immobilization. (d). Rinse the electrode gently with PBS to remove unbound enzyme. (e). Store the prepared biosensor at 4 °C when not in use.

## 3. Results and Discussion

### 3.1. Mechanism of Ti_3_C_2_T_x_ MQDs-Based Electrochemical Cholinesterase-Inhibiting Biosensor for OP Pesticides

**Figure 1:**
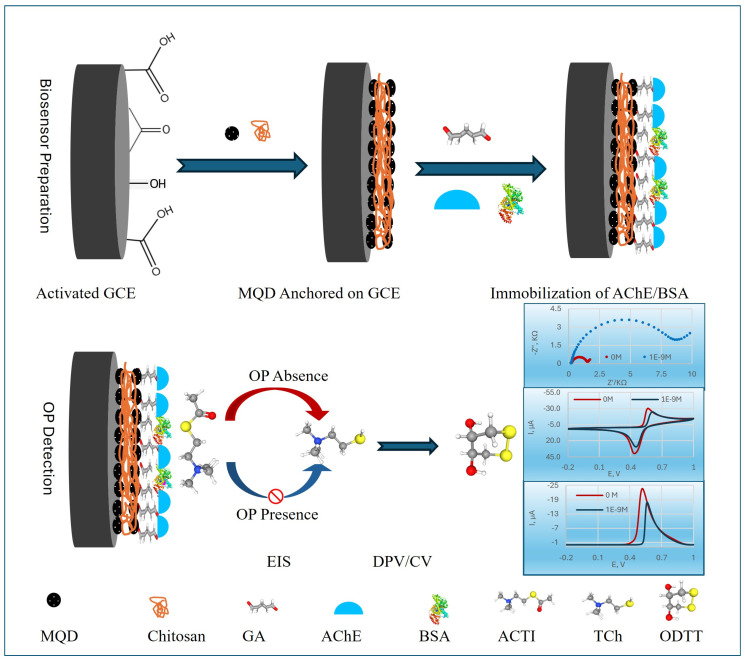
Schematic illustration of the MQDs-based electrochemical OP biosensor.

Figure 1 illustrates the comprehensive mechanism of the MQDs-based electrochemical AChE biosensor designed for the detection of OP pesticides. The fabrication of this biosensor involves a precise sequence of steps. First, the GCE surface was meticulously cleaned and then electrochemically activated. This activation process enhanced the electrode’s surface area and improved electron transfer kinetics, creating an optimal foundation for subsequent modifications.

Next, MQDs were anchored onto the electrochemically activated GCE surface using CS. CS, a biocompatible biopolymer, acts as a stable matrix, facilitating the uniform dispersion and robust immobilization of the MQDs, which are crucial for enhancing electron transfer and signal amplification, and preventing the peeling of MQDs from the sensor surface directly ensures the long-term stability and reliable performance of the prepared biosensor [43].

Then, AChE and BSA were co-crosslinked onto the MQDs-modified surface using GA. This crosslinking created a stable, three-dimensional network that firmly entrapped the AChE enzyme, preventing its leaching and preserving its catalytic activity, while BSA provided a protective and biocompatible microenvironment [44].

Finally, the detection mechanism relies on the catalytic activity of the immobilized AChE. In the absence of OP, AChE efficiently hydrolyzed ACTI into electroactive thiocholine (TCh).

AChE catalyzes hydrolysis:

$${\left[CH_{3}CO-S-CH_{2}-CH_{2}-N{\left(CH_{3}\right)}_{3}\right]}^{+}+\;H_{2}O\overset{AChE}{\to }[HS-CH_{2}-CH_{2}-N(C{H_{3})}_{3}{]}^{+}$$

So, the key electroactive species generated is TCh ([HS–CH_2_–CH_2_–N(CH_3_)_3_]^+^).

At the biosensor surface, TCh undergoes oxidation of its thiol group (-SH) and produces thiocholine disulfide (TCh–TCh):

$$2[HS-CH_{2}-CH_{2}-N(C{H_{3})}_{3}{]}^{+}\to [-S-CH_{2}-CH_{2}-N^{+}(C{H_{3})}_{3}{]}_{2}+2H^{+}+2e^{-}$$

The resulting TCh produced a strong electrochemical signal, measured by DPV, EIS, or CV. When the biosensor was exposed to OPs, these compounds inhibited the activity of AChE. This inhibition led to a reduced production of TCh, which in turn caused a quantifiable decrease in the DPV signal, allowing for the sensitive and precise determination of pesticide concentration.

### 3.2. Synthesis and Characterization of Ti_3_C_2_T_x_ MXene and Ti_3_C_2_T_x_ MQDs

**Figure 2:**
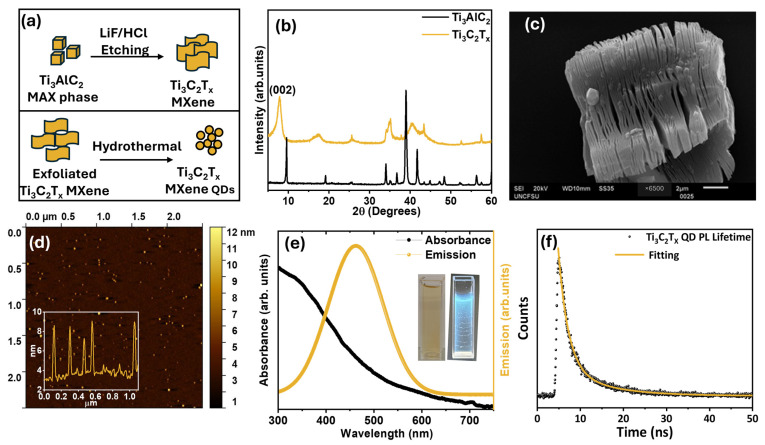
(**a**) Schematic representation showing the transformation from MAX phase to Ti_3_C_2_T_x_ MXene and subsequently to Ti_3_C_2_T_x_ MQDs; (**b**) X-ray diffraction (XRD) patterns of the Ti_3_AlC_2_ MAX phase and the corresponding Ti_3_C_2_T_x_ MXene 2D sheets after etching; (**c**) scanning electron microscopy (SEM) image of layered Ti_3_C_2_T_x_ MXene sheets, (**d**) atomic force microscopy (AFM) image depicting the morphology of Ti_3_C_2_T_x_ MQDs; (**e**) UV–Vis absorbance and photoluminescence (PL) emission spectra of the synthesized MQDs (insets are MQD solution under visible light and 365 nm UV lamp), (**f**) photoluminescence (PL) lifetime profile of the Ti_3_C_2_T_x_ MQDs.

Figure 2a provides a schematic overview of the preparation process for Ti_3_C_2_T_x_ MXene and Ti_3_C_2_T_x_ MQDs. We began with the Ti_3_AlC_2_ MAX phase, which was etched using the LiF/HCl method to synthesize Ti_3_C_2_T_x_ MXene. The resulting MXene was first characterized by XRD, as shown in Figure 2b. The XRD pattern displays a distinct peak near 9°, indicating successful etching of the MAX phase. Additionally, the SEM image (Figure 2c) reveals a layered sheet-like structure, further confirming the formation of MXene sheets. These sheets were then exfoliated and processed to prepare MQDs. The topography image of AFM in Figure 2d displayed the nanoscale dimensions of the particles (height: 1.1–10.8 nm; mean: 6.4 nm), following into the QD range. This was further confirmed by the inset line profile of the produced MQDs which are below 10 nm, validating the successful formation of MQDs. Optical properties, UV–Vis absorbance and PL emission spectra were recorded. Figure 2e reveals that the MQDs have absorbance in the range of 300 to 450 nm and emission between 350–650 nm. Figure 2f presents the PL decay profile of the MQDs solution, which fits a biexponential model with lifetimes τ_1_= 1.98 ns and τ_2_ = 8.37 ns and corresponding amplitudes A_1_ = 735 and A_2_ = 259, respectively. The calculated average lifetime (τ_avg_) is approximately 5.8 ns. The shorter component τ1, with the higher amplitude, is attributed to dominant radiative recombination of excitons or shallow trap states, while the slower component τ_2_ is attributed to trap-assisted recombination via defects or functional groups [45].

### 3.3. Analytical Performance of Electrochemical Cholinesterase-Inhibiting Biosensor for OP Pesticides

The integration of MQDs with AChE yielded a highly sensitive electrochemical biosensor for OP detection. A widely used OP insecticide, CPS, was tested as the model inhibitor for the following reasons: First, its significant health and environmental impact, including neurotoxicity and genotoxicity, underscores the critical need for effective detection methods, making it a highly relevant target for study [46,47]. Second, its extensive global application in agriculture and pest control leads to its frequent presence as an environmental contaminant [48]. Especially, the chemical structure of CPS is representative of the phosphorothioate class of OP insecticides, and it is a potent AChE inhibitor, binding to and phosphorylating the enzyme, which forms the core detection principle for these biosensors.

**Figure 3:**
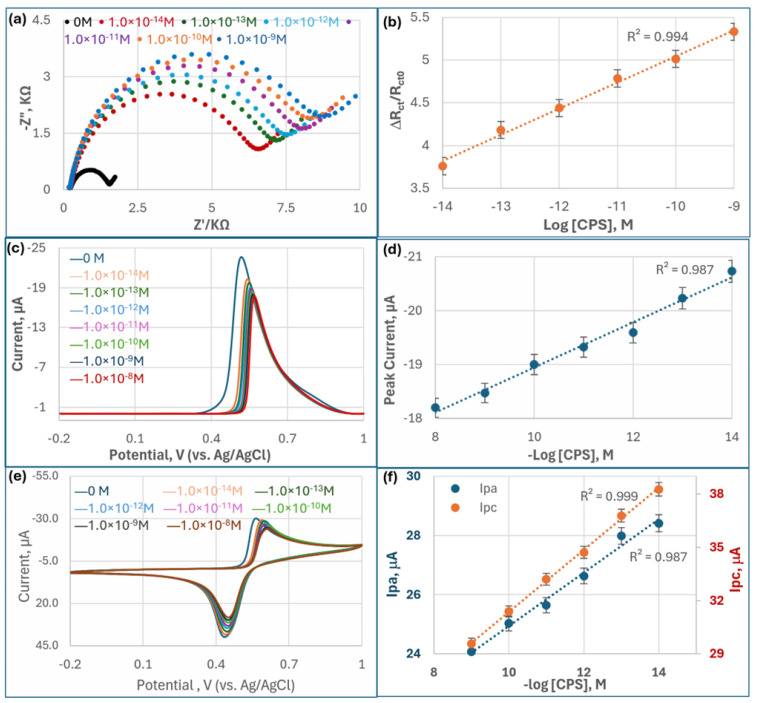
Dynamic linear ranges of electrochemical cholinesterase-inhibiting biosensor for OP pesticides in PBS buffer at 25 °C. All other working conditions follow Section 2.3. Electrochemical Measurement. (1). Nyquist plots from electrochemical impedance spectroscopy (EIS) of the MQD-AChE biosensor for increasing concentrations of CPS (**a**) and the linear relationship between normalized charge-transfer resistance and *log*[CPS] (**b**). (2). Differential pulse voltammetry (DPV) responses of the biosensor to CPS (**c**) and the linear relationship between peak current and *log*[CPS] (**d**). (3). Cyclic voltammograms (CV) (scan rate: 25 mV/s) for CPS (**e**) and the linear relationship between anodic peak current (Ipa, Blue) and cathodic peak current (Ipc, Orange) with *log*[CPS] (**f**).

**Figure 4:**
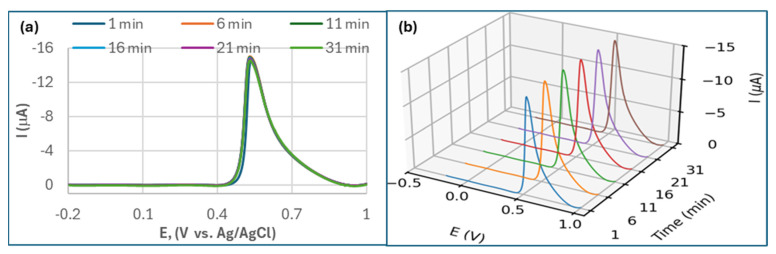
Repeated DPV scan of MQD-AChE biosensor in the presence of 1 × 10^−9^ M of CPS concentration at 1, 6, 11, 16, 21, and 31 min. (**a**) is the overlay view and (**b**) is the 3D view.

After the electrochemical biosensor was exposed to CPS for 10 min, the inhibition of AChE caused a dramatic increase in the charge transfer resistance in EIS, a decrease in the anodic peak of TCh in DPV and CV, and a decrease in the cathodic peak in CV compared to that without pesticide inhibition. The biosensor was thus characterized using EIS, DPV, and CV, with each technique providing complementary insights into the detection mechanism, sensitivity (Figure 3), and stability (Figure 4). OP pesticide inhibition of AChE depends on time for the pesticide to phosphorylate the serine residue at the enzyme’s active site. For biosensor essays, time-dependent optimization is necessary. For example, a smartphone-integrated biosensor for OP optimized incubation and found that the inhibition effect plateaued at 10 min, balancing sensitivity and assay throughput [5]. Thus, 10 min was selected as an optimal compromise to balance sensitivity, specificity, and practical assay duration.

The electrochemical biosensor quantifies OP inhibitors by measuring the inhibition of AChE activity. The biosensor’s function relies on the enzymatic hydrolysis of the substrate ATCI by immobilized AChE. This reaction yields TCh, an electroactive product that is oxidized at a specific potential, generating a measurable anodic current peak. The presence of an OP inhibitor in the sample inhibits the catalytic activity of AChE. This inhibition reduces the rate of TCh production, resulting in a proportional decrease in the observed anodic current. The degree of current inhibition is directly correlated with the concentration of the OP inhibitor.

The relationship between inhibition and concentration can be described by the following equation [49,50]:

(1) $$I=I_{0}(1-\frac{\left[OP\right]}{K_{i}+\left[OP\right]})$$

where *I* is the inhibited current, *I*_0_ is the uninhibited current, [*OP*] is the concentration of the OP inhibitor, and *K_i_* is the inhibition constant.

This equation can be simplified into the following equation:

(2) $$I=I_{0}\frac{K_{i}}{K_{i}+[OP]}$$

As OP concentration increases, the fraction of enzyme inhibited follows a saturation-like, nonlinear trend, not by a simple proportionality. At low OP concentrations, small increases result in significant inhibition. At the OP concentration, [*OP*] << *K_i_*, the peak current *I* is linear to [*OP*], while at high concentrations, enzyme inactivation saturates, causing smaller incremental changes. As a result, the sensor current, reflecting residual AChE activity, decreases rapidly at low OP concentrations, and plateaus at higher concentrations. This nonlinear relationship transforms into a linear response when plotted as current versus the logarithm of OP concentration, making it easier to quantify OP over several orders of magnitude, a wider range of concentrations, enhancing the accuracy and dynamic range of the quantitative analysis. At an extremely high concentration, [*OP*] >> *K_i_*, the peak current *I* is linear to *1*/[*OP*]. *log I* and *log* [*OP*] is linearly related.

Utilizing DPV data, the inhibition constant (*K_i_*) for the AChE–CPS interaction on the MQD-based biosensor surface was estimated to be 62 nM by fitting the data to the non-linear inhibition equation using a Solver function in Excel.

#### 3.3.1. EIS Analysis

Figure 3a presents Nyquist plots illustrating the EIS response of the AChE-based biosensor upon exposure to varying concentrations of CPS. Each curve consists of a semicircular portion followed by a linear segment. The diameter of the semicircle, corresponding to the charge transfer resistance (*Rct*), increases progressively with rising CPS concentrations. This indicates that as more CPS molecules inhibit AChE activity on the electrode surface, the enzyme’s ability to hydrolyze the substrate (ACTI) into the electroactive product (TCh) is reduced. The decreased production of this electroactive species disrupts the redox reactions at the electrode surface, resulting in higher impedance. The curves demonstrate clear separation, confirming that the biosensor responds sensitively and discriminatively to different CPS concentrations, with larger semicircles reflecting greater inhibition.

Interestingly, the EIS results show a pronounced difference between the blank sample (0 M OPs) and all other CPS concentrations, highlighted by the smallest semicircle in the Nyquist plot for the blank. This momentous change indicates that, in the absence of CPS compounds, the AChE enzyme retains full catalytic activity, facilitating rapid electron transfer between the electroactive species and the electrode surface. As soon as CPS is introduced, even at very low concentrations, it inhibits AChE by phosphorylating the active site serine residue, dramatically reducing enzymatic activity. This inhibition impairs the hydrolysis of the substrate and disrupts redox reactions, thereby increasing *R_ct_*. The sharp increase in *R_ct_* after exposure to even trace levels of CPS underscores the biosensor’s high sensitivity and effective signal transduction, making it especially suitable for early detection of CPS contamination.

**Figure A1:**
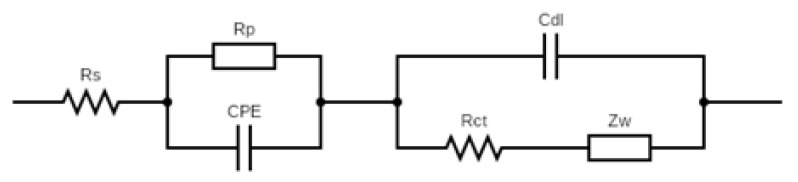
The equivalent circuit of the MQD-based OP biosensor for the EIS simulation.

Reliable *R_ct_* values were quantified by fitting impedance spectra to an equivalent circuit model (Figure A1). To normalize electrode-to-electrode variations, the relative change in charge-transfer resistance was calculated as follows:

$$\frac{∆R_{ct}}{R_{ct0}}=\frac{R_{ct}-R_{ct0}}{R_{ct0}}$$

where *R_ct_* and *R_ct_*_0_ represent the charge transfer resistance in the presence of and absence of the inhibitor, respectively.

Figure 3b demonstrates a robust linear relationship (R^2^ = 0.99) between the normalized impedance change $\left(\frac{∆R_{ct}}{R_{ct0}}\right)$ and the logarithm of CPS concentration, *log*[CPS], across a linear dynamic range of 10^−14^–10^−9^ M, a very high sensitivity and range for CPS determination. The linear relationship observed in Figure 3b underscores the reliability and quantitative capability of the MQD-based OP biosensor for chlorpyrifos detection. The strong correlation (R^2^ = 0.99) between the normalized charge transfer resistance change $\left(\frac{∆R_{ct}}{R_{ct0}}\right)$ and the logarithm of CPS concentration indicates that the impedance response is not random but systematically proportional to the degree of AChE inhibition. This linearity is critical for establishing accurate calibration curves, enabling the sensor to quantify unknown concentrations with high precision. Furthermore, the wide linear dynamic range (10^−14^–10^−9^ M) confirms that the biosensor can sensitively capture both trace and environmentally relevant levels of CPS. Compared with conventional biosensors, which often suffer from nonlinearity at low or high inhibitor concentrations due to enzyme saturation or weak signal-to-noise ratios, the MQD-based design ensures consistent signal transduction [6]. Thus, the strong linearity demonstrated in Figure 3b not only validates the biosensor’s sensitivity but also highlights its suitability for real-world environmental monitoring, where reproducibility and accuracy across a broad range of pesticide concentrations are essential.

#### 3.3.2. DPV Analysis

DPV measurements (Figure 3c) revealed a distinct decrease in the oxidation peak current as CPS concentrations increased, resulting from reduced electroactive product (TCh) generation upon AChE inhibition. Figure 3d demonstrates a strong linear correlation between peak current and *log*[CPS] (R^2^ = 0.99) across a broader range (10^−14^–10^−8^ M) compared to the results of EIS, with an exceptionally low detection limit of 1 × 10^−17^ M. The narrower linear range of EIS techniques likely arises from saturation effects at high CPS concentrations around 10^−8^ M, where complete AChE inhibition limits further resistance changes. DPV outperformed EIS and CV (Figure 3e,f) in sensitivity and linear range due to its pulse-based design, which minimizes non-Faradaic currents (e.g., capacitive background) and amplifies Faradaic signals, thereby substantially enhancing the signal-to-noise ratio of the measurement [51]. This enhanced signal-to-noise ratios, especially at ultralow concentrations. The visual clarity of the DPV peaks observed in the presented graphs, characterized by sharp peaks and a relatively flat baseline, provided direct empirical evidence of DPV’s effectiveness in enhancing signal quality. This clean signal is paramount for accurate and reliable quantification of enzyme inhibition, especially when attempting to detect subtle changes induced by low concentrations of inhibitors.

#### 3.3.3. CV Analysis

CV scans (Figure 3e) showed decreasing anodic (*I_pa_*) and cathodic peak currents (*I_pc_*) with higher CPS concentrations. While both *I_pa_* and *I_pc_* exhibited linear dependence on *log*[CPS] (Figure 3f), *I_pc_* displayed superior sensitivity (R^2^ = 0.999) compared to *I_pa_* (R^2^ = 0.987). This anomaly probably arises from the reaction mechanism: *I_pa_* corresponds to the oxidation of TCh (the enzymatic product), which is susceptible to interference from oxygen or electrode fouling, reducing reproducibility. On the other hand, *I_pc_* reflects the reduction of disulfide bonds (e.g., oxidized dithiothreitol, ODTT), a process less affected by background noise. The cleaner cathodic baseline and reversible redox behavior of thiol groups yield higher precision and sensitivity for *I_pc_*. The linear dynamic range was found to be 10^−14^–10^−9^ M, comparable to the EIS results.

Comparing the three techniques, DPV seems to be the optimal choice for quantitative CPS detection due to its unmatched detection limit (10^−17^ M), wide linear range, and resistance to non-Faradaic interference. CV provides mechanistic insights but suffers from higher signal variability, while EIS is limited by saturation at high concentrations. EIS measurement is typically slower than DPV and CV, which is another characteristic to be considered. However, by using normalized charge-transfer resistance $\left(\frac{∆R_{ct}}{R_{ct0}}\right)$, EIS may reduce the variations in sensitivity between different biosensors raised from electrode fabrication inconsistencies.

In comparison with other nanomaterials-based OP electrochemical biosensors reported in the literature, our MQD-based OP biosensor showed enhanced performance for OP detection (Table 1) [14,17,52,53,54]. The superior performance of the Ti_3_C_2_T_x_ MQD-based biosensor is attributed to the integration of nanoscale engineering, robust enzyme immobilization, and optimized electrode interfaces. This design leverages several structural and material advantages, such as the following: (1). High surface-to-volume ratio: MQDs (mean diameter 6.4 nm) provide a much larger surface area compared to MXene nanosheets, offering abundant sites for enzyme (AChE/BSA) immobilization and enhancing catalytic efficiency. (2). Quantum confinement and edge-rich surfaces: MQDs expose more catalytically active edge sites, which improve electron transfer and electrochemical signal quality, a critical feature for sensitive detection. (3). Superior conductivity: The ultra-small size of MQDs facilitates rapid electron shuttling between the enzyme and the electrode, increasing the biosensor’s sensitivity to OP compounds [55].

Our MQD-based biosensor also demonstrated outstanding performance compared to other QD-based electrochemical biosensors for OP compounds. Tanwar et al. developed an electrochemical OP biosensor using graphene quantum dots (GQDs) to measure malathion concentrations ranging from 1 to 30 μM. Using DPV, they achieved a limit of detection of 0.62 nM [60]. In another study, Yadav et al. recently reported a detection limit of 0.01 ppm (30 nM) for malathion by modifying a screen-printed electrode with silica nanoparticles (SiO_2_) and GQDs using square wave voltammetry [14]. The superior performance of the MQD-based biosensor may be attributed to the key advantages of MQDs, including their favorable biocompatibility, ease of functionalization, hydrophilicity, stability, and lower toxicity [32,33,34,35].

In brief, the MQD-AChE electrochemical biosensor is a promising platform for fast OP detection, combining exceptional sensitivity and broad linearity. DPV is the preferred technique for ultra trace analysis with minimized interference from non-Faradaic currents. In the meantime, *I_pc_* in CV offers a reliable alternative for mechanistic studies.

#### 3.3.4. The MQD-AChE Biosensor Offered Operational Stability for OP Detection

The biosensor showed a good stability, with less than 3.5% relative variance of its initial DPV response after 31 min of continuous operation at fixed CPS concentrations (Figure 4). In contrast, we developed an OP biosensor based on Ti_3_C_2_T_x_ MXene nanosheets using an identical fabrication procedure. This MXene-based inhibition biosensor also exhibited a detection range of 10^−14^ to 10^−8^ M for CPS. However, it demonstrated suboptimal linearity (R^2^ < 0.90) and a much lower analytical sensitivity due to the high standard deviation, and the sensor suffered from rapid delamination of the MXene layers from GCE surface.

The superior sensing stability first benefits from the design for stabilization and biocompatibility: (1). Strong enzyme anchoring: GA cross-linking covalently stabilizes AChE-BSA complexes, preventing enzyme leaching and denaturation while maintaining bioactivity [61,62]. (2). BSA as a protective layer: BSA acts as a spacer and protective layer, reducing enzyme denaturation and enhancing stability [44]. (3). CS Matrix: CS disperses MQDs evenly and provides amino groups for cross-linking, combining biocompatibility with high conductivity for efficient signal amplification [43].

Electrochemical activation of GCE also optimized the electrode surface. Electrooxidation treatment in H_2_SO_4_ solution generates carboxylic/carbonyl groups on GCE surface, which improved hydrophilicity and adhesion for the MQD-CS composite, leading to more stable and uniform coatings [63].

More importantly, unlike larger MXene sheets, MQDs do not aggregate in CS, ensuring even coatings and consistent electron transfer. Thinner MQD films maintain a robust interface with the GCE, minimizing delamination during repeated electrochemical cycling [55]. In addition, MQDs form less dense films, allowing the substrates ACTI to diffuse rapidly to AChE’s active sites, improving sensitivity and detection speed [64].

The combined use of electrochemically activated GCE, MQD-CS matrix, and cross-linked AChE/BSA results in a biosensor with exceptional sensitivity and stability. The quantum confinement effects and edge-rich surfaces of MQDs are particularly effective for enzyme coupling and signal transduction, making this approach superior to biosensors based on larger MXene structures or other nanomaterials.

Nevertheless, this study used the drop-casting method to prepare the biosensor. This may cause inconsistencies in electrode fabrication due to manual deposition variability, coffee-ring effect [65], and enzyme activity loss due to repeated use or washing, indicating weak attachment and inconsistent enzyme activity. We have noticed the inconstancy of the sensor signal and absolute electrode resistance variation between different batches of biosensors. However, there is not significant difference in linear range and detection limits. Future work will test the inkjet printing method to achieve more uniform enzyme deposition, leading to higher reproducibility and superior electrochemical performance compared to traditional drop-cast electrodes [66].

#### 3.3.5. The MQD-AChE Biosensor Applies to Other Cholinesterase-Inhibiting OP Pesticides

**Figure 5:**
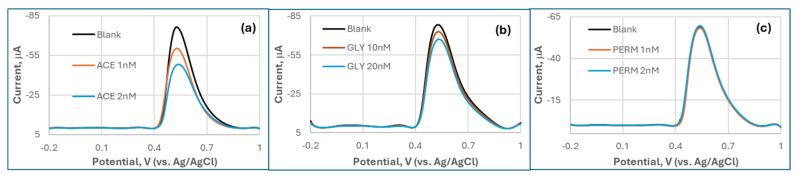
DPV response of the MQD-based biosensor by adding (**a**). acephate (ACE); (**b**). glyphosate (GLY); (**c**). a negative control, permethrin (PERM).

In addition to CPS, two other OPs (ACE and GLY) and a pyrethroid negative control, PERM, were also tested with the MQD-based biosensor (Figure 5). DPV analysis demonstrated distinct responses to OPs versus non-target neurotoxins. Although they are weaker than CPS, both ACE and GLY induced significant concentration-dependent suppression of the oxidation peak current at ~0.65 V, corresponding to TCh oxidation. ACE exhibited the suppression response (21% at 1 nM; 37% at 2 nM) and GLY (7% at 10 nM; 14% at 20 nM), with GLY showing additional peak broadening. In stark contrast, PERM, a pyrethroid negative control, produced negligible signal changes (<5% at 1–2 nM) without peak shifts, confirming its lack of AChE inhibition. OP compounds collectively generated >5-fold higher current suppression than PERM, highlighting the biosensor’s selectivity for AChE inhibitors.

Not surprisingly, the biosensor’s selectivity arises from the specific biochemical interaction between OPs and AChE. CPS, ACE, and GLY irreversibly phosphorylate AChE’s catalytic serine residue, inhibiting enzymatic hydrolysis of ACTI and reducing electroactive TCh production [48]. This directly suppresses the DPV oxidation current proportionally to OP concentration and inhibition kinetics. Conversely, PERM’s null response validates its non-cholinergic mechanism (sodium channel modulation), eliminating false positives.

The distinct electrochemical signatures, sharp suppression for high-affinity CPS versus broadening for GLY, further enable differentiation of OP subclasses. CPS and ACE are both organophosphate insecticides that directly inhibit AChE by phosphorylating the enzyme’s active site, leading to the accumulation of acetylcholine and overstimulation of cholinergic pathways; however, CPS is generally more potent due to its rapid conversion to the highly active CPS oxon, whereas ACE is considered a weak inhibitor of AChE, the greater insecticidal potency and mammalian toxicity of which are largely due to its metabolic activation to methamidophos [48,67,68]. GLY, in contrast, is not an organophosphate herbicide that does not efficiently bind or inhibit AChE at biologically relevant concentrations, showing only weak and reversible inhibition at high concentration levels, and its neurotoxic effects are mainly attributed to oxidative stress rather than cholinergic disruption [69,70,71]. The P-C bond in GLY is much more stable and less prone to hydrolysis than the P-O-C bonds in CPS and ACE. This difference is largely responsible for the fact that GLY is a much weaker cholinesterase inhibitor compared to many organophosphate insecticides. PERM, a pyrethroid insecticide, targets voltage-gated sodium channels and causes neuronal hyperexcitation; while it can indirectly influence cholinergic activity by disrupting calcium signaling and neurotransmitter release, it does not directly inhibit AChE except at very high doses or in combination with other neurotoxicants [72,73]. Thus, the different inhibition effects on cholinergic activity among these compounds are primarily due to their distinct chemical structures, mechanisms of action, and molecular affinities for AChE, with CPS and ACE exerting direct inhibition and GLY and PERM showing minimal or only indirect effects. By excluding pyrethroids while detecting diverse OPs, the biosensor offers a reliable screening tool for environmental OP contamination with 5 min response times and nM to sub-fM sensitivity.

### 3.4. Application of MQD-Based OP Biosensor to Monitor In Vitro Cholinergic Activity for Bean Beetles

**Figure A2:**
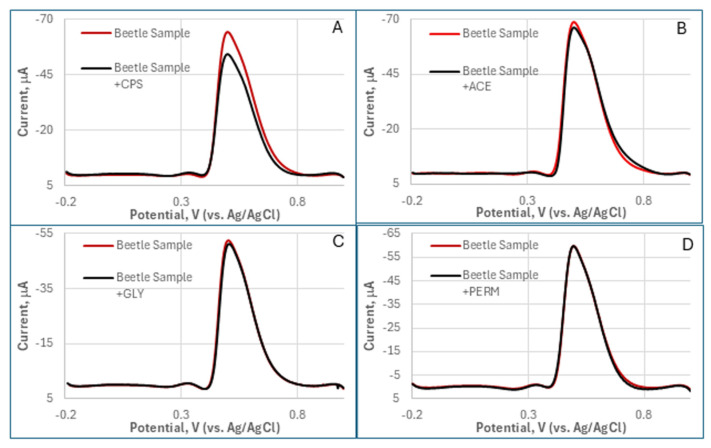
DPV responses of the MQD-based OP biosensor in homogenized beetle sample solution showed the distinctive inhibition effect for OPs ((**A**): CPS, (**B**): ACE, and (**C**): GLY) compared to the control ((**D**): PERM).

The MQD-based OP biosensor was employed to assess the cholinergic inhibitory effects of the above four distinct pesticides (CPS, ACE, GLY, and PERM) on AChE activity within *Callosobruchus maculatus* (bean beetle) homogenates. Analyzing complex biological matrices like insect homogenates necessitates careful consideration of matrix effects [74]. Endogenous compounds, including natural metabolites, lipids, and proteins, can interfere with enzymatic activity or signal generation, potentially leading to deviations from results obtained in buffered solutions. While a discernible matrix effect on biosensor sensitivity was noted in comparison to the earlier test in PBS buffer (Section 3.3), distinct DPV responses were obtained for each pesticide (Figure A2). The magnitude of current decrease, indicative of AChE inhibition, was highest for CPS, became weaker for ACE, was extremely lowe for GLY, and there was an undetectable change for PERM (the control).

**Figure A3:**
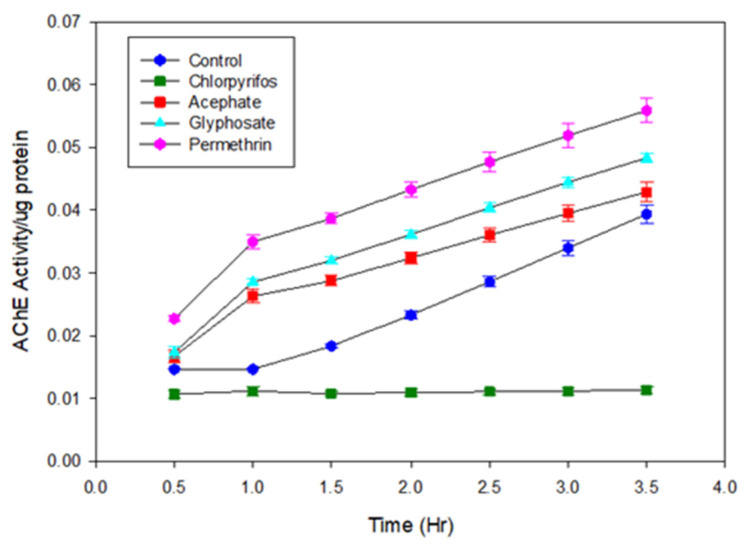
In vitro cholinergic activity assay of CPS (green), ACE (red), GLY (cyan), PERM (magenta), and the negative control (blue) in homogenate of bean beetles.

For comparative analysis, the conventional Ellman colorimetric assay was utilized to evaluate cholinergic activity in bean beetle homogenates. As depicted in Figure A3, the Ellman assay results demonstrate the impact of CPS, ACE, GLY, and PERM on cholinergic activity over a 3.5 h period. The negative control group (untreated beetles) exhibited a steady, moderate increase in activity, representing baseline cholinergic function. In contrast, the CPS treatment group showed minimal to no increase in activity throughout the assay, maintaining near-zero values, which signifies potent inhibition of cholinergic activity, showing a strong consistency for CPS between the MQD-based biosensor and the conventional Ellman method.

The ACE group did not demonstrate a significant reduction in activity compared to the negative control in the Ellman colorimetric assay. While ACE is known to inhibit acetylcholinesterase, it is a weak and reversible inhibitor and must be metabolized to methamidophos to increase inhibition. The short incubation time might have been a factor in the activation of ACE [75]. Conversely, both GLY and PERM treatments resulted in elevated activity levels relative to the negative control, with PERM eliciting the highest increase among all treatments. GLY-treated samples also displayed elevated activity, albeit slightly less pronounced than PERM. These results showed the challenge to Ellman colorimetric assay for the detection of weaker inhibitors at low concentrations (60 µg/mL in this experiment).

In contrast, the observed current changes in the biosensor correlate directly with AChE activity, highlighting the potential applicability of this biosensor in biological samples. The MQD-based OP biosensor presents several notable advantages over the conventional Ellman method. These include enhanced sensitivity and lower detection limits, reduced sample preparation, and faster response times. It can be used for both strong and weak inhibition at very low concentrations. Furthermore, its portability and suitability for field deployment offer a significant advantage over the Ellman method, which typically necessitates laboratory infrastructure and a spectrophotometer. As an electrochemical biosensor, it directly converts the biochemical event (TCh oxidation) into a quantifiable electrical signal, facilitating seamless integration with digital systems for data processing and analysis. Future investigations will focus on refining the biosensor’s performance to enhance sensitivity and selectivity across a broader spectrum of complex real-world matrices, including diverse insect species, various agricultural products, and environmental water samples. Comprehensive understanding and mitigation of matrix effects and synergistic inhibitions are critical for ensuring robust biosensor performance [76].

## 4. Conclusions

An electrochemical biosensor for the detection of OPs was successfully developed using a composite of AChE/CS/Ti_3_C_2_T_x_ MQDs, which enabled effective immobilization of the AChE enzyme. The development of the Ti_3_C_2_T_x_ MQD-based electrochemical biosensor for OP pesticide detection represents a significant advancement in environmental monitoring and biological sample analysis. The meticulous fabrication process, involving the electrochemical activation of a GCE surface, stable immobilization of MQDs with CS, and co-crosslinking of AChE and BSA, yielded a robust and highly sensitive platform. Comparative analysis of electrochemical techniques demonstrated that DPV is the optimal method for quantitative OP detection with this biosensor, offering an unmatched detection limit of 1 × 10^−17^ M for CPS, a wide linear range (10^−14^–10^−8^ M), and superior resistance to non-Faradaic interference. While CV provided valuable mechanistic insights, and EIS offered insights into charge transfer resistance, DPV’s enhanced signal-to-noise ratio proved critical for ultra-trace analysis.

The superior performance and operational stability of the MQD-AChE biosensor are attributed to the unique properties of the MQDs, including their high surface-to-volume ratio, quantum confinement effects, edge-rich surfaces, and excellent conductivity, which facilitate efficient enzyme immobilization and electron transfer. The design also benefits from strong enzyme anchoring via GA cross-linking, the protective role of BSA, and the biocompatible and conductive CS matrix. The use of electrochemically activated GCE further contributes to stable and uniform coatings, minimizing delamination issues observed with larger MXene sheets.

Crucially, the biosensor demonstrated excellent selectivity for AChE-inhibiting OPs (CPS, ACE, and GLY) over non-cholinergic neurotoxins like PERM, providing a reliable screening tool with rapid response times and high sensitivity. Its successful application in monitoring in vitro cholinergic activity in bean beetle homogenates, with results strongly correlating with the conventional Ellman method, underscores its potential for real-world biological sample analysis.

Future efforts will focus on improving the consistency of biosensor fabrication, potentially through inkjet printing methods, to enhance reproducibility. The promising attributes of this MQD-based biosensor, including its enhanced sensitivity, lower detection limits, reduced sample preparation, faster response times, and portability, position it as a highly valuable tool for comprehensive understanding and mitigation of OP contamination in diverse complex matrices.

## Figures and Tables

**Table 1:** The MQD-based biosensor described in this work demonstrates superior performance for organophosphorus (OP) pesticide detection when compared to other nanomaterial-based electrochemical biosensors reported in the literature.

Nanomaterial Used.	Target OP	DL (M)	Linear Range (M)
MQDs	CPS	1 × 10^−17^	10^−14^ to 10^−8^
Pt/MoS2/Ti_3_C_2_ MXene nanosheets [26]	CPS	4.71 × 10^−13^	10^−12^ to 10^−6^
MXene/AuPt nanocomposite [56]	CPS	4.4 × 10^−15^	3 × 10^−11^ to 3 × 10^−6^
Ag NPs–CGR–NF [57]	CPS	5.3 × 10^−14^	1.0 × 10^−13^ to 1 × 10^−8^
AuPd nanoparticle/carbon nanotube/MXene [58]	CPS	1.98 × 10^−12^	2.85 × 10^−12^ to 2.85 × 10^−6^
Polyvinyl alcohol/azide-unit water-pendant/Fe-Ni nanoparticle [59]	Phosmet	1 × 10^−10^	1 × 10^−10^–5 × 10^−9^
SiO_2_ and Graphene QDs [14]	Malathion	2.3 × 10^−12^	10^−11^ to 10^−6^

## Data Availability

The data are contained within the article.

## Appendix A. EIS Simulation and CV Analysis

### Circuit for EIS Simulation

In the equivalent circuit of the MQD-based OP biosensor (Figure A1), each element represents a physical or electrochemical process at the electrode–electrolyte interface. The charge transfer resistance (Rct), which is our target, represents the resistance to electron transfer between the electrode surface and the redox mediator, which increases upon AChE inhibition by OP pesticides. The double-layer capacitance (C_dl_) reflects the capacitive behavior arising from charge separation at the electrode–electrolyte interface. The Warburg impedance (Zw) models the diffusion of electroactive species to and from the electrode surface. The solution resistance (Rs) corresponds to the ionic resistance of the electrolyte. A constant phase element (CPE) accounts for non-ideal capacitive behavior due to surface roughness or heterogeneity of the MQD-based OP biosensor. Finally, the polarization resistance (Rp) represents the additional resistance from AChE biolayer polarization and non-Faradaic processes at the GCE interface. Together, these components describe the interfacial processes governing the impedance response of the MQD-based OP biosensor.

## Appendix B. MQD-Based OP Biosensor to Monitor In Vitro Cholinergic Activity for Bean Beetles

The cholinergic inhibitory effects of chlorpyrifos (CPS), acephate (ACE), glyphosate (GLY), and permethrin (PERM) on acetylcholinesterase (AChE) activity in bean beetle (Callosobruchus maculatus) homogenates were tested using the MQD-based biosensor and compared to in vitro colorimetric assay based on the Ellman method.

### Appendix B.1. Cholinergic Activity Monitored by MQD-Based Electrochemical Biosensor

First, 1 mL homogenized beetle sample was first mixed with 9 mL of PBS and 1 mM of ATI. Differential pulse voltammetry (DPV) data were collected before and after adding 1 nM pesticides (CPS, ACE, GLY, and PERM) for 10 min (Figure A2).

### Appendix B.2. In Vitro Cholinergic Activity Assay in Bean Beetles

At least 10 adult bean beetles per treatment group were sacrificed and homogenized in 1 mL of ice-cold phosphate buffer (0.1 M, pH 6.8), followed by centrifugation (15,000× *g*, 5 min) to obtain enzyme-containing supernatants with protein concentrations standardized to 0.5–1.0 mg/mL. Pesticides (CPS, ACE, GLY, and PERM) and the negative control (deionized water) were tested by pre-incubating 50 μL of homogenate with 50 μL of pesticide solution (final OP concentration: 60 μg/mL) for 30 min at 30 °C, after which a master mix containing phosphate buffer and 5,5′-dithiobis-(2-nitrobenzoic acid) (DTNB) (0.45 mM) was added. The reaction was initiated with acetylthiocholine iodide (ACTI, 1.0 mM) in 96-well plates (total volume: 200 μL), and AChE activity was measured spectrophotometrically at 412 nm over 3.5 h.

BSA Standard: Before running the assays, a standard Bovine Serum Albumin (BSA) was used to determine total protein in each sample. Total protein was determined using a modified Bradford assay. This standard protein assay involved using BSA as the standard. For each sample, approximately 10–20 μL of sample supernatant was used to measure protein content. The Tecan Infinite 200 Pro Multimode Microplate Reader was used to measure absorbance at 595 nm. The amount of absorbance was proportional to the amount of protein present in the sample.

Activity was calculated using the thiocholine extinction coefficient and normalized to protein content, revealing pesticide-specific inhibitory effects compared to the negative control (Figure A3).
